# Lipids bearing extruded-spheronized pellets for extended release of poorly soluble antiemetic agent—Meclizine HCl

**DOI:** 10.1186/s12944-017-0466-x

**Published:** 2017-04-12

**Authors:** Faaiza Qazi, Muhammad Harris Shoaib, Rabia Ismail Yousuf, Muhammad Iqbal Nasiri, Kamran Ahmed, Mansoor Ahmad

**Affiliations:** 1 Department of Pharmaceutics, Faculty of Pharmacy & Pharmaceutical Sciences, University of Karachi, Karachi, 75270 Pakistan; 2 Research Institute of Pharmaceutical Sciences, Department of Pharmacognosy, Faculty of Pharmacy & Pharmaceutical Sciences, University of Karachi, Karachi, 75270 Pakistan

**Keywords:** Meclizine HCl, Pellets, Lipids, Extended release, Extrusion spheronization

## Abstract

**Background:**

Antiemetic agent Meclizine HCl, widely prescribed in vertigo, is available only in immediate release dosage forms. The approved therapeutic dose and shorter elimination half-life make Meclizine HCl a potential candidate to be formulated in extended release dosage form. This study was aimed to develop extended release Meclizine HCl pellets by extrusion spheronization using natural and synthetic lipids. Influence of lipid type, drug/lipid ratio and combinations of different lipids on drug release and sphericity of pellets were evaluated.

**Methods:**

Thirty two formulations were prepared with four different lipids, Glyceryl monostearate (Geleol^®^), Glyceryl palmitostearate (Precirol^®^), Glyceryl behenate (Compritol^®^) and Carnauba wax, utilized either alone or in combinations of drug/lipid ratio of 1:0.5–1:3. Dissolution studies were performed at variable pH and release kinetics were analyzed. Fourier transform infrared spectroscopy was conducted and no drug lipid interaction was found.

**Results:**

Sphericity indicated by shape factor (e_R_) varied with type and concentration of lipids: Geleol^®^ (e_R_ = 0.891–0.997), Precirol^®^ (e_R_ = 0.611–0.743), Compritol^®^ (e_R_ = 0.665–0.729) and Carnauba wax (e_R_ = 0.499-0.551). Highly spherical pellets were obtained with Geleol^®^ (Aspect ratio = 1.005–1.052) whereas irregularly shaped pellets were formed using Carnauba wax (Aspect ratio = 1.153–1.309). Drug release was effectively controlled by three different combinations of lipids: (i) Geleol^®^ and Compritol^®^, (ii) Geleol^®^ and Carnauba wax and (iii) Geleol^®^, Compritol^®^ and Carnauba wax. Scanning electron microscopy of Compritol^®^ pellets showed smooth surface with pores, whereas, irregular rough surface with hollow depressions was observed in Carnauba wax pellets. Energy dispersive spectroscopy indicated elemental composition of lipid matrix pellets. Kinetics of (i) Geleol^®^ and Compritol^®^ pellets, explained by Korsmeyer-Peppas (R^2^ = 0.978–0.993) indicated non-Fickian diffusion (*n* = 0.519-0.597). Combinations of (ii) Geleol^®^ and Carnauba wax and (iii) Geleol^®^, Compritol^®^ and Carnauba wax pellets followed Zero-order (R^2^ = 0.991–0.995). Similarity test was performed using combination of Geleol^®^ and Compritol^®^ (i) as a reference.

**Conclusions:**

Matrices for the extended release of Meclizine HCl from extruded-spheronized pellets were successfully formed by using three lipids (Geleol^®^, Compritol^®^ and Carnauba wax) in different combinations. The encapsulated pellets of Meclizine HCl can be effectively used for treatment of motion sickness, nausea and vertigo for extended period of time.

## Background

Meclizine HCl is a histamine (H_1_) receptor antagonist, indicated for prophylactic treatment and management of vomiting, nausea and dizziness due to motion sickness. It is also indicated for pruritus, anaphylactic reactions and vertigo associated with diseases affecting the vestibular system (e.g., Meniere disease, labyrinthitis) [1, 2]. It is more frequently prescribed because of fewer adverse effects [1]. It ranks among the top 200 drugs of 2014 as per total prescription count [3]. It is currently available as immediate release tablets, capsules and chewable tablets [1]. It is not available as an extended release (ER) dosage form. Literature survey results show that lipid based ER pellets of Meclizine HCl have not been reported yet. Meclizine HCl has a plasma elimination half-life of 5–6 h [1, 2]. For the treatment of vertigo, the standard dose is 25–100 mg/day, given in two or more divided doses [4]. ER formulation of Meclizine HCl is thus, required to enhance patient compliance while reducing dosing intervals and minimizing risk of dose related adverse effects.

In comparison to monolithic system, pellets provide added benefits of multiple unit dosage forms. They are rapidly dispersed in gastrointestinal tract and enhance drug absorption and bioavailability. Use of pellets reduce peak plasma fluctuations and minimize risk of dose dumping. Pellet formulations also have provision of further modifications [5, 6]. Clinical studies demonstrate that pellets provide flexibility in doses by variations in the amount of administered drug [6]. Different techniques are available for manufacturing of pellets such as layering of drug cores in a fluid bed coater, extrusion spheronization, melt pelletization and direct pelletization in a rotary processor or high shear mixer. Although, melt granulation is the most commonly reported method for pelletization of lipids [7–11] but it may lead to drug stability issues because the powder mass is melted at high temperature [12]. Extrusion spheronization method followed by wet granulation, is preferred for the preparation of extended release pellets. This technique is reported to be the most robust and reproducible means for the preparation of multiparticulates (pellets) with spherical shape, high density, low friability, high drug loading capacity and good flow properties in a reasonable time [6, 13].

In oral drug delivery system, lipids are successfully used to enhance swallowability, shelf life, provision of modified release profiles, taste masking, reduction of gastric irritation and to improve bioavailability of poorly soluble drugs [14]. Meclizine HCl, a Biopharmaceutics classification system (BCS) class II agent, is attributed with low solubility and high permeability [15]. Increased bioavailability and prolonged drug delivery of Meclizine HCl can be achieved by preparation of lipids bearing extruded-spheronized pellets because of the resemblance of lipids to in vivo components and its unique set of physiochemical properties [16].

Glyceryl monostearate, GMS (Geleol^®^), Glyceryl palmitostearate, GPS (Precirol^®^), and Glyceryl behenate, GB (Compritol^®^) are three of the lipid glycerides, which form lipid matrices owing to their high melting points and are recommended for sustained delivery of drugs [17]. They are reported to be effective release retardants in ER dosage forms [7–10, 16]. GMS (Geleol^®^) is a mono glyceride of stearic acid having two free hydroxyl groups [10]. GPS (Precirol^®^) is a combination of glycerides (mono, di and tri) of palmitic acid and stearic acid. GB (Compritol^®^) is a mixture of mono, di and tri behenate of glycerol, prepared by the esterification of behenic acid (C22) with glycerine. GPS (Precirol^®^) and GB (Compritol^®^) have low HLB value (2), indicating marked hydrophobicity of these two glycerides due to the absence of polyethylene glycol esters and esterification of glycerol by long chain fatty acids [8]. Another promising lipid excipient to extend the release is Carnauba wax [14]. Carnauba wax is mainly composed of esters of carboxylic acid (C24 and C28) and straight chain primary alcohols (C32 and C34) [18]. It controls drug release through pore diffusion and erosion when applied as a coating polymer or used as a matrix former [5, 18–20].

The objective of this study was to formulate ER pellets of Meclizine HCl by extrusion spheronization method using matrices of GMS (Geleol^®^), GPS (Precirol^®^), GB (Compritol^®^) and Carnauba wax. The influence of lipid type, drug/lipid ratio and combination of lipids on drug release and sphericity of pellets were evaluated. Drug excipient interaction was determined by Fourier transform infrared (FTIR) spectroscopy. Elemental characterization was carried out by Energy dispersive spectroscopy (EDS) along with Scanning electron microscopy (SEM) to reveal the surface morphology of pellets. In vitro dissolution was performed at variable pH (1.2, 4.5 and 6.8) and release profiles were evaluated using different kinetic models. Accelerated stability was also conducted for optimized formulations.

## Materials and methods

### Materials

Meclizine HCl was a gift from Ali Gohar Pharmaceuticals Private Limited (Pakistan). Lipids: GMS (Geleol^®^ 40–55, type I), GPS (Precirol^®^ATO5), GB (Compritol^®^888ATO) were obtained from Gattefosse Foundation (Saint-Priest, France). CW was provided by BDH laboratory suppliers (England). Microcrystalline Cellulose, MCC (Avicel PH-101) was purchased from FMC Corporation (USA). Freshly prepared distilled water (DW) was used and all analytical grade reagents and solvents were utilized.

## Methods

### Dose calculation

The dose for ER Meclizine HCl was calculated from the given formula:

1 $$ {D}_t= Dose\ \left(1+0.693\times \raisebox{1ex}{$ t$}\!\left/ \!\raisebox{-1ex}{${t}_{\raisebox{1ex}{$1$}\!\left/ \!\raisebox{-1ex}{$2$}\right.}$}\right.\right) $$

Where, *D*_*t*_ is total dose, *t* is time for extended drug release i.e. 12 h and *t*_*1/2*_ is the drug half-life [21]. Thus, by applying the above equation, extended release dose of Meclizine HCl is found to be 60 mg.

### Experimental design

Different blends of Meclizine HCl (60 mg) were prepared using MCC (spheronizing aid), Geleol^®^, Precirol^®^, Compritol^®^ and CW (release controlling lipids). The experiments were conducted in four groups: Group 1 based on single lipid; Group 2: combination of two lipids; Group 3: combination of three lipids; Group 4: combination of four lipids. Thirty two formulations were designed. These formulations were based on drug/lipid ratio of 1:1–1:3 except Group 1 in which the drug/lipid ratio was 1:0.5–1:2. MCC concentration was fixed to provide identical plasticity upon extrusion and brittleness during spheronization (Table 1). Group 1 formulations were prepared for initial screening of four different lipids (Geleol^®^, Precirol^®^, Compritol^®^ and CW) and their individual effect on release of Meclizine HCl and sphericity of pellets. Drug/lipid ratio of 1:0.5–1:2 was selected from relevant published studies [18, 22, 23]. Further Groups were designed on the basis of Group 1 findings in which drug/lipid ratio was increased to 1:3 and combination of lipids were used [19, 24].

**Table 1:** Composition of 60 mg Meclizine HCl ER matrix pellets formulations

Groups	Codes	Drug: Lipid Ratio	Geleol®		Precirol®		Compritol®		Carnauba Wax		MCC		Granulating Fluid	Total wt.
			mg	%	mg	%	mg	%	mg	%	mg	%	%	mg
Group 1: Single Lipids	F1	1:0.5	30	20	-	-	-	-	-	-	60	40	39	150
	F2	1:1	60	30	-	-	-	-	-	-	80	40	33	200
	F3	1:2	120	40	-	-	-	-	-	-	120	40	30	300
	F4	1:0.5	-	-	30	20	-	-	-	-	60	40	44	150
	F5	1:1	-	-	60	30	-	-	-	-	80	40	39	200
	F6	1:2	-	-	120	40	-	-	-	-	120	40	33	300
	F7	1:0.5	-	-	-	-	30	20	-	-	60	40	43	150
	F8	1:1	-	-	-	-	60	30	-	-	80	40	41	200
	F9	1:2	-	-	-	-	120	40	-	-	120	40	33	300
	F10	1:0.5	-	-	-	-	-	-	30	20	60	40	41	150
	F11	1:1	-	-	-	-	-	-	60	30	80	40	33	200
	F12	1:2	-	-	-	-	-	-	120	40	120	40	28	300
Group 2: Two Lipids Combinations	F13	1:1	20	10	60	30	-	-	-	-	60	30	35	200
	F14	1:2	20	10	100	50	-	-	-	-	20	10	29	200
	F15	1:3	30	10	180	60	-	-	-	-	30	10	26	300
	F16	1:1	20	10	-	-	60	30	-	-	60	30	35	200
	F17	1:2	20	10	-	-	100	50	-	-	20	10	29	200
	F18	1:3	30	10	-	-	180	60	-	-	30	10	24	300
	F19	1:1	20	10	-	-	-	-	60	30	60	30	27	200
	F20	1:2	20	10	-	-	-	-	100	50	20	10	23	200
Group 3: Three Lipids Combinations	F21	1:1	20	10	20	10	20	10	-	-	80	40	31	200
	F22	1:2	40	20	40	20	40	20	-	-	20	10	27	200
	F23	1:3	60	20	60	20	60	20	-	-	60	20	22	300
	F24	1:1	20	10	20	10	-	-	20	10	80	40	27	200
	F25	1:2	40	20	40	20	-	-	40	20	20	10	22	200
	F26	1:3	60	20	60	20	-	-	60	20	60	20	20	300
	F27	1:1	20	10	-	-	20	10	20	10	80	40	29	200
	F28	1:2	40	20	-	-	40	20	40	20	20	10	26	200
	F29	1:3	60	20	-	-	60	20	60	20	60	20	20	300
Group 4: Four Lipids Combinations	F30	1:1	15	8	15	8	15	8	15	8	80	40	24	200
	F31	1:2	30	15	30	15	30	15	30	15	20	10	19	200
	F32	1:3	45	15	45	15	45	15	45	15	60	20	17	300

### Preparation of extended release pellets

Lipids were pulverized in a mortar and pestle. Hard CW was difficult to grind due to its plastic nature. Drug and excipients were weighted after passing through 40 mesh sieve (American Society of Testing and Materials, ASTM) and were blended dry for 10 min in a planetary mixer. Mixed powder was granulated using DW. Quantity of DW was adjusted based on preliminary experiments to achieve the spherical pellets and maximum yield. DW was poured gradually and wet mixing was continued until homogenous and cohesive mass was achieved. Uniform water distribution was ensured by repeatedly scrapping sides of the bowl. The wet mass was immediately processed with laboratory screw extruder (Caleva Process Solution Ltd., UK) fitted with screen (1 mm), operated at 50–60 rpm. Extrudes were collected in a tray, broken down manually into small cylinders and then spheronized (Caleva Process Solution Ltd., UK) for 10 min at 600–800 rpm. The wet pellets were dried in an oven for 12 h at 40 °C [13]. Dried screened pellets were filled into 0 size hard gelatin capsule shells.

### Fourier transform infrared spectroscopy (FTIR)

Pure drug and optimized formulations were subjected to IR spectroscopy using FTIR spectrophotometer (Thermo Nicolet Avatar, 330) to determine interactions between active drug and excipients. Spectra were scanned from 4000 to 500 cm-1 wave number using OMNIC™ Spectra Software.

### Moisture content and size of pellets

Water content of pellets immediately after spheronization and drying were determined in each batch. Samples were placed on petri dishes and heated to 40 °C in a hot air oven until the moisture content (MC) became constant. Pellets were sieved using sieve shaker containing nest of standard sieves for 10 min. Pellets retained on each sieve were weighed. Size in the range of 800–1500 μm was considered appropriate and utilized for further studies.

### Flow properties of pellets

Forty gram pellets from each batch were placed in a 100 ml measuring cylinder. Initial and tapped volumes were recorded. Static angle of repose was determined by measuring height of symmetrical cone of pellets formed through a funnel at a fixed base. Mean and standard deviation of three readings were used.

2 $$ Bulk\  density=\frac{M}{V_o} $$

3 $$ Tapped\  density=\frac{M}{V_f} $$

4 $$ Compressibility\  Index=\left(\frac{V_o-{V}_f}{V_o}\right)\times 100 $$

5 $$ Hausner\  ratio=\frac{V_o}{V_f} $$

6 $$ {tan}_{\left(\propto \right)}=\frac{height}{0.5\  base} $$

Where, M is the mass of pellets, V_o_ and V_f_ are the bulk and tapped volumes of pellets respectively. Compressibility index 11–15 and hausner ratio 1.12–1.18 show good flow properties, whereas, compressibility index ≤10 and hausner ratio 1.00–1.11 exhibit excellent flow properties. Angle of repose shows good flow from 31 to 35 and excellent flow from 25 to 30 [25].

### Friability of pellets

10 g pellets were placed in a friabilator wheel (Erweka GmbH D-63150, Husenstamm, Germany) and subjected to falling shocks at 25 rpm for 4 min. 250 μm mesh was used to remove fines and the friability was calculated by remaining above fraction.

7 $$ Friability\ \left(\%\right)=\frac{\left( Initial\  Weight- Final\  Weight\right)}{Initial\  Weight}\times 100 $$

Friability less than 1% is considered acceptable. Each batch was analysed thrice [26].

### Shape and area of pellets

Shape and area of each pellet batch (*n* ≥ 50) was evaluated using stereomicroscope (Am Scope Digital, LED-1444A, USA). The digitalized images were further analysed by image analysis software (NIH Image J 1.47v, USA). Area, perimeter, feret diameter were measured and shape factors were calculated as follows:

8 $$ {d}_{ce}=\sqrt{\frac{4 A}{\pi}} $$

9 $$ \mathrm{Aspect}\ \mathrm{ratio}\ \left(\mathrm{AR}\right)={\mathrm{d}}_{\max }/{\mathrm{d}}_{\min } $$

10 $$ Circularity\ (C)=4\pi A/{P}^2 $$

11 $$ Shape\  factor\ \left({e}_R\right)=\frac{2\pi}{P}\frac{r_e}{f}-\sqrt{1-{\left(\frac{b}{l}\right)}^2} $$

12 $$ Correction\  factor\ (f)=1.008-0.231\left(1-\frac{b}{l}\right) $$

Where*, d*_*ce*_ is the circle equivalent diameter, *A* is the area, *d*_*min*_ and *d*_max_ are the shortest and longest feret diameters respectively, *P* is the perimeter, *e*_*R*_ is the two dimensional shape factor, *r*_*e*_ is the mean radius, *f* is a correction factor, *l* and *b* are length and breadth of the pellet respectively. *b* = *l* are round pellets, *b*˂*l* are elliptical pellets having an aspect ratio 1.2–1.5. The limiting value is 1.1 for aspect ratio, whereas, the acceptable lower limit value for e_R_ is 0.6 [27, 28].

### Surface morphology and elemental characterization of pellets

External morphology of pellets were visualized using Scanning electron microscope, SEM (JSM-6380A, Jeol, Japan) at 10 kV. Whole pellet was placed on aluminium studs and sputter coated with gold up to 250°A by means of an Auto Coater (JFC-1500, Jeol, Japan). Photomicrographs were obtained at magnification ranging from 50 to 1500 times. Elements were characterized using Energy Dispersive Spectrometer (EDS) attached with SEM at 20 kV accelerated voltage.

### Drug content analysis

Twenty capsules from each batch were randomly selected. The capsule contents were pulverized by means of mortar and pestle. 10 μg/ml sample solution was prepared by utilizing mean weight equivalent quantity in mobile phase containing 1.5 g of sodium 1-heptanesulfonate in mixture of DW (300 ml) and acetonitrile (700 ml) at pH 4 (adjusted with 0.1 N Sulfuric acid). The samples were sonicated, filtered and then injected. Signals were detected at 230 nm. Assay was carried out using C18 column (25 cm × 4.6 mm) with 5 μm packing on HPLC (LC-10AT VP, No.C20973806986 LP, Shimadzu Corporation, Kyoto, Japan). Mean and standard deviation of three readings from each batch were used [25].

### In vitro drug release study

Meclizine HCl release was determined using USP Apparatus 1 six station (Erweka DT600, Husenstamm, Germany) in 0.01 N HCl (900 ml) maintained at 37 ± 0.5 °C at 100 rpm. 10 ml dissolution samples were drawn at each 1 h interval over 12 h. Sink condition was maintained by immediately replenished volumes with fresh medium. Collected samples were filtered and finally diluted to attain suitable concentration. Samples were analysed at 230 nm on spectrophotometer (UV-1800, Double beam Spectrophotometer, No.A11454500172CD, Shimadzu Corporation, Kyoto, Japan). Cumulative drug release (percentage) was determined and plotted against time (hours). Six samples (mean of each batch) were used [29].

### Drug release kinetic studies

#### Model-dependent methods

Various kinetic models including Zero-order (Eq. (13)), First-order (Eq. (14)), Higuchi square root (Eq. (15)), Hixson-Crowell cube root (Eq. (16)), Baker-Lonsdale (Eq. (17)), Jander’s equation (Eq. (18)) and Korsmeyer-Peppas model (Eq. (19)) were applied to in vitro release data of Meclizine HCl to determine its release kinetics using MS Excel (DD Solver).

13 $$ {Q}_t={k}_0 t $$

14 $$ \mathit{\log}{Q}_t=\mathit{\log}{Q}_0+{k}_1\frac{t}{2.303} $$

15 $$ {Q}_t={k}_H{t}^{\raisebox{1ex}{$1$}\!\left/ \!\raisebox{-1ex}{$2$}\right.} $$

16 $$ \sqrt[3]{Q_0}-\sqrt[3]{Q_t}={k}_{HC} t $$

17 $$ \raisebox{1ex}{$3$}\!\left/ \!\raisebox{-1ex}{$2$}\right.\left[1-{\left(1-\raisebox{1ex}{${M}_t$}\!\left/ \!\raisebox{-1ex}{${M}_{\infty }$}\right.\right)}^{\raisebox{1ex}{$2$}\!\left/ \!\raisebox{-1ex}{$3$}\right.}\right]-\raisebox{1ex}{${M}_t$}\!\left/ \!\raisebox{-1ex}{${M}_{\infty }$}\right.={k}_{BL} t $$

18 $$ 1-{\left(1-\raisebox{1ex}{${M}_t$}\!\left/ \!\raisebox{-1ex}{${M}_{\infty }$}\right.\right)}^{\raisebox{1ex}{$1$}\!\left/ \!\raisebox{-1ex}{$3$}\right.}={k}_J{t}^{\raisebox{1ex}{$1$}\!\left/ \!\raisebox{-1ex}{$2$}\right.} $$

19 $$ \raisebox{1ex}{${M}_t$}\!\left/ \!\raisebox{-1ex}{${M}_{\infty }$}\right.= k{t}^n $$

where *Q*_*t*_ is the amount of drug released in time *t*, *Q*_*0*_ is the initial amount of drug in the sample, *M*_*t.*_ is the amount of drug released in time *t*, *M*_∞_ is the amount at infinite time, *M*_*t.*_ */M*_∞_ is the fractional solute release, *t* is the time in h, *t*_*1/2*_ is the square root of time and *K*_*0*_ *, K*_*1*_ *, K*_*H*_ *, K*_*HC*_ *, K*_*BL*_ *, K*_*J*_ and *K* are the release rate constants for Zero-order, First-order, Higuchi, Hixson-Crowell cube root, Baker-Lonsdale, Jander’s equation and Korsmeyer-Peppas model respectively. *n* is an exponent which characterizes the different release mechanisms and calculated through slope of the straight line [30].

Drug release was further characterized by determining the mean dissolution time (MDT) and dissolution efficiency (DE) using following equations:

20 $$ MDT=\frac{\sum_{j=1}^n{\widehat{t}}_j\varDelta {M}_j}{\sum_{j=1}^n\varDelta {M}_j} $$

21 $$ D. E=\frac{\underset{0}{\overset{t}{\int }} y\times dt\ }{y_{100}\times t}\times 100 $$

Where *j* is the sample number, *n* is the number of dissolution sample times, ${\widehat{t}}_j$ is the time at midpoint between *t*_*j*_ and *t*_*j-1*_, ∆M_j_ is the additional amount of drug dissolved between *t*_*j*_ and *t*_*j-1*_ and *y* is the drug dissolved (percentage) at time *t* [31].

#### Model-independent method (dissolution profile comparison)

Similarity in dissolution profiles was compared by determining the Similarity factor (*f*_2_):

22 $$ {f}_2=50\times \mathit{\log}\left\{{\left[1+\left(\frac{1}{n}\right){\sum}_{t-1}^n{\left({R}_t-{T}_t\right)}^2\right]}^{-0.5}\times 100\right\} $$

Where *R*_*t*_ and *T*_*t*_ are the amount of drug released from the reference and test formulations at each time point, respectively, *n* is the number of dissolution samples. Release profiles are considered different if *f*_2_ < 50 [30].

#### Stability studies

Optimized ER pellets were studied at 40 ± 2 °C/75% ± 5% RH (relative humidity) for accelerated stability for 6 months, in line with guidelines of International Conference on Harmonisation (ICH). Encapsulated pellets were placed in amber glass bottles and stored in humidity chamber (Nuaire, USA). Samples were drawn every 3 months and their drug content, physical appearance and release characteristics in different media were determined. Software Stab (R-Gui, version 3.1.1) was used to calculate the shelf life.

## Results

### Influence of lipids

Initial trial formulations in Group 1 were prepared using single lipid in order to evaluate its effect on drug release and sphericity. Three formulations were prepared with each lipid in drug/lipid ratio of 1:0.5–1:2 as shown in Table 1. Although, MCC serves as an extrusion aid in concentrations less than 20%, but in this study amount of MCC was kept constant at 40% in order to prolong the release of poorly soluble Meclizine HCl.

### Effect of Geleol^®^ (GMS)

**Fig. 1:**
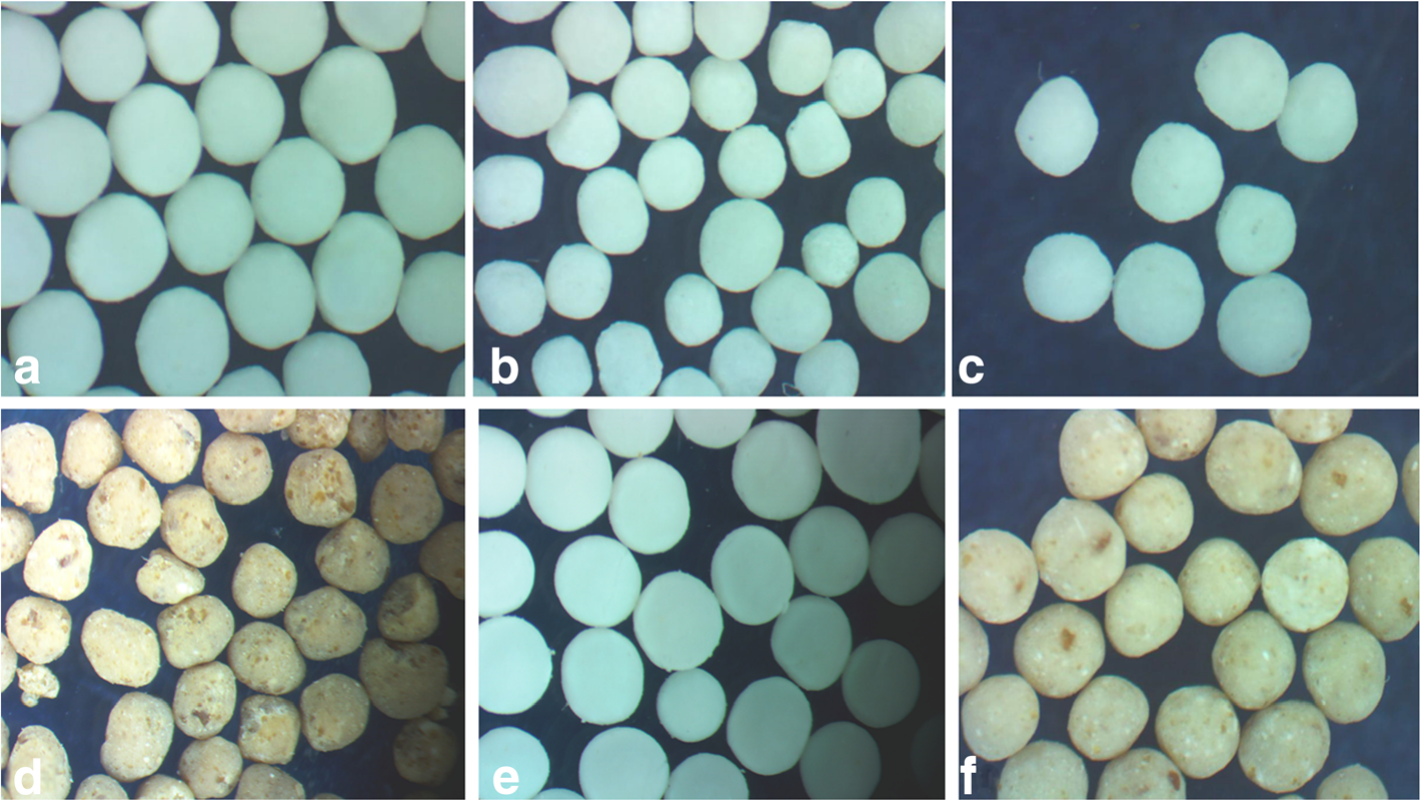
Stereo images of Meclizine HCl matrix pellets prepared with (**a**) Geleol^®^ (**b**) Precirol^®^ (**c**) Compritol^®^ (**d**) CW (**e**) Geleol^®^ and Compritol^®^ (**f**) Geleol^®^, Compritol and CW

**Fig. 2:**
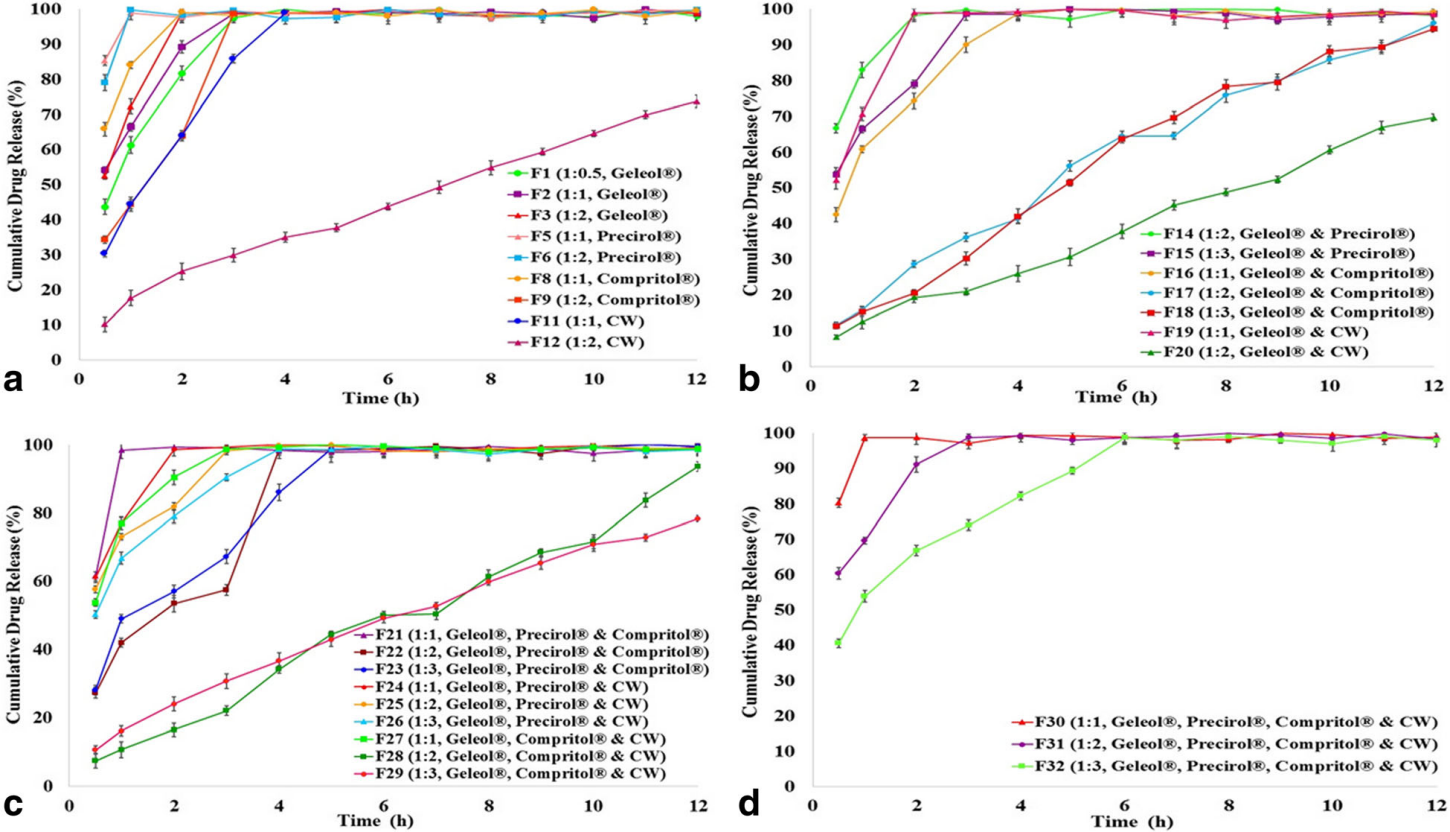
In vitro release profiles of Meclizine HCl matrix pellets showing effect of (**a**) single lipid (**b**) combinations of two lipids (**c**) combinations of three lipids (**d**) combinations of four lipids

Large and highly spherical pellets (Table 1) were prepared by using Geleol^®^ (GMS) which utilized 30-39% granulating fluid, illustrated in Fig. 1(a). Highest aspect ratio (1.005) and shape factor (0.997) were obtained in Geleol^®^ pellets in drug/lipid ratio of 1:2. Amount of granulating fluid was decreased with increased drug/lipid ratio (Table 1). Release of Meclizine HCl was rapid from Geleol^®^ pellets (F1-F3), shown in Fig. 2(a).

### Effect of Precirol^®^ (GPS)

Small and nearly spherical pellets (Table 1), were formulated by using Precirol^®^ (GPS) which required, 33–44% granulating fluid, shown in Fig. 1(b). Aspect ratio and shape factor of Precirol^®^ pellets ranged from 1.027–1.047 and 0.611–0.743 respectively. Meclizine HCl was rapidly released from Precirol^®^ pellets (F4-F6). Precirol^®^ pellets having drug/lipid ratio 1:2, illustrated in Fig. 2(a) released 90% drug within 2 hours.

### ***Effect of Compritol***^®^ (GB)

F7-F9 comprised of Compritol^®^ pellets which utilized 33–43% granulating fluid during preparation of pellets. Pellets were large and almost spherical having aspect ratio (1.017–1.058) and two dimensional shape factor (0.665–0.729), shown in Fig. 1(c). Release of Meclizine HCl was decreased as the concentration of Compritol^®^ was increased, illustrated in Fig. 2(a).

### Effect of carnauba wax

CW formed large and irregularly shaped pellets, even in presence of 40% MCC, shown in Fig. 1(d). Lowest aspect ratio (1.309) and shape factor (0.499) were obtained in CW pellets. As concentration of CW was increased in the matrix pellets, aspect ratio was also increased but shape factor was decreased. Release of Meclizine HCl from CW pellets showed inverse relationship (F10-F12). F12 released only 73% drug over a period of 12 h, illustrated in Fig. 2(a).

### Effect of MCC

MCC formed spherical pellets of Meclizine HCl with Geleol^®^, Precirol^®^ and Compritol^®^. When it was used with CW irregularly shaped pellets were formed, shown in Fig. 1(d). This irregularity in shape was more pronounced with increased concentration of CW. MCC (40%) had no effect on release of Meclizine HCl.

### Effect of combinations of two lipids

Results of Group 1 indicated that Geleol^®^ formed highly spherical pellets, compared to other lipids. Therefore, Geleol^®^ was added in each formulation of Group 2 by replacing some amount of MCC to produce round pellets. Group 2 combinations, (i). Geleol^®^ and Compritol^®^ and (ii) Geleol^®^ and CW produced retarding effect on drug release. Geleol^®^ and Compritol^®^ formed highly spherical and round pellets, shown in Fig. 1(e). Release of Meclizine HCl was successfully sustained by combination of Geleol^®^ and Compritol^®^ at drug/lipid ratio of 1:2 (F17) and 1:3 (F18). F17 and F18 released 90% drug within 11 and 12 h respectively, shown in Fig. 2(b). Combination of Geleol^®^ and CW at drug/lipid ratio of 1:1 (F19) and 1:2 (F20) also produced spherical shaped pellets. F19 (1:1) released 90% drug within 2 h. F20 (1:2) released 69% drug which indicated a pronounced retardation in drug release, shown in Fig. 2(b).

### Effect of combinations of three lipids

In Group 3, Geleol^®^ and two other lipids were combined to evaluate cumulative influence on drug release and sphericity. Group 3 pellets were spherical (Fig. 1(f)) with acceptable aspect ratios and shape factors (Table 2). In Group 3, the combination of Geleol^®^, Compritol^®^ and CW in drug/lipid ratio of 1:2 (F28) and 1:3 (F29), effectively prolonged the drug release up to 12 h. Combination of Geleol^®^, Compritol^®^ and CW in drug/lipid ratio of 1:3 (F29) released 78% drug at the end of 12 h, indicating excessive control on release of Meclizine HCl, shown in Fig. 2(c).

**Table 2:** Image analysis of Meclizine HCl ER matrix pellets formulations

Codes	Area	Perimeter	Circularity	Feret diameter (μm)	dce	Aspect Ratio	eR
F1	10,900	390.771	0.897	121.696	117.836	1.016	0.970
F2	12,522	411.942	0.927	133.765	126.300	1.052	0.891
F3	14,304	438.502	0.935	140.357	134.988	1.005	0.997
F4	8729	360.185	0.846	111.879	105.450	1.047	0.611
F5	8801	355.000	0.878	110.725	105.884	1.027	0.743
F6	10,349	376.124	0.919	119.641	114.819	1.043	0.712
F7	10,044	383.563	0.858	119.436	113.115	1.017	0.665
F8	10,424	377.838	0.918	121.840	115.234	1.058	0.700
F9	12,868	417.443	0.928	133.544	128.033	1.042	0.729
F10	13,464	438.623	0.879	143.171	130.964	1.153	0.551
F11	13,513	431.267	0.913	150.881	131.202	1.234	0.548
F12	14,422	444.383	0.918	155.003	135.543	1.309	0.499
F13	10,773	384.07	0.918	125.543	117.148	1.073	0.675
F14	11,553	393.681	0.937	130.648	121.314	1.093	0.675
F15	12,366	411.898	0.916	136.015	125.510	1.069	0.854
F16	12,143	405.315	0.929	133.989	124.374	1.068	0.735
F17	12,758	415.931	0.927	133.417	127.484	1.092	0.750
F18	14,773	461.71	0.871	143.809	137.183	1.050	0.970
F19	11,504	395.95	0.922	125.873	121.057	1.031	0.718
F20	12,573	412.134	0.93	139.119	126.557	1.083	0.840
F21	12,830	432.91	0.86	134.358	127.843	1.055	0.758
F22	14,213	437.057	0.935	141.209	134.558	1.028	0.773
F23	14,952	463.925	0.873	147.146	138.011	1.063	0.988
F24	10,819	395.937	0.867	124.808	117.397	1.066	0.801
F25	12,720	412.587	0.939	133.989	127.294	1.052	0.772
F26	13,067	419.126	0.935	133.135	129.0188	1.046	0.726
F27	12,477	409.367	0.936	132.412	126.0725	1.039	0.737
F28	13,576	427.222	0.935	138.004	131.5077	1.069	0.721
F29	14,992	448.414	0.937	144.7	138.1958	1.028	0.838
F30	11,517	404.16	0.886	130.973	121.1253	1.098	0.796
F31	11,343	397.655	0.901	125.196	120.2068	1.007	0.768
F32	12,884	418.726	0.923	134.302	128.1122	1.041	0.882

### Effect of combinations of four lipids

In Group 4, all four lipids were combined (F30-F32) to determine effects on drug release and sphericity. The combination of four lipids resulted in formation of spherical pellets (Table 2) with rapid drug release, illustrated in Fig. 2(d).

### Drug excipient interactions

**Fig. 3:**
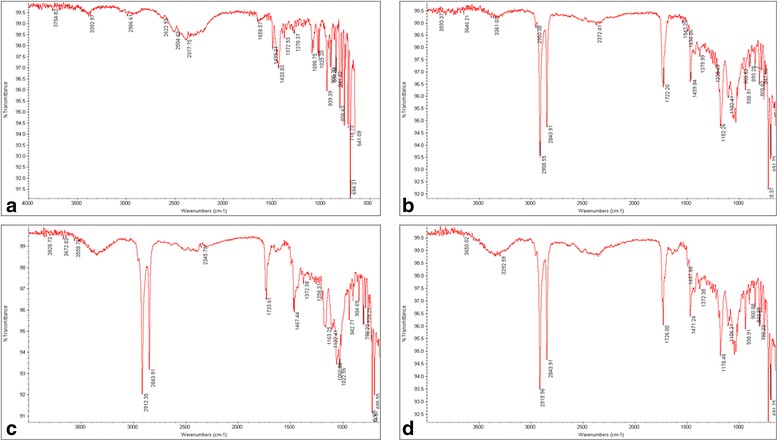
FTIR spectra showing (**a**) pure Meclizine HCl (**b**) combination of Geleol® and Compritol^®^ in drug/lipid ratio of 1:2 (F17) (**c**) combination of Geleol^®^ and CW in drug/lipid ratio of 1:2 (F20) (**d**) combination of Geleol^®^, Compritol^®^ and CW in drug/lipid ratio of 1:2 (F28)

Characteristic peaks of pure drug at 2986.61 cm^−1^ (C-H str.), 1658.57 cm^−1^ (C═C str.), 1499.21 cm^−1^ (CH2 bending), 1433.83 cm^−1^ (CH3), 1270.37 cm^−1^ (C-N str.), 718.78 cm^−1^ (C-Cl str.) are illustrated in Fig. 3(a). FTIR spectra of optimized formulations of F17 (Combination of Geleol^®^ and Compritol^®^), F20 (Combination of Geleol^®^ and CW) and F28 (Combination of Geleol^®^, Compritol^®^ and CW) are shown in Fig. 3(b), (c) & (d) respectively, indicating absence of any drug lipid interactions.

### Flow properties and friability of pellets

Table 3 shows rheological properties of formulations. Results of compressibility index, hausner ratio and angle of repose indicate excellent flow properties for all lipids (Geleol^®^, Precirol^®^ and Compritol^®^) of Group 1 except CW. Combination of CW with other lipids in Group 2, Group 3 and Group 4 resulted in pellets with satisfactory flow properties. Friability of all pellet formulations was adequate, indicating that pellets were strong enough to bear attrition and shock during transportation, consumption and storage.

**Table 3:** Physicochemical characteristics of Meclizine HCl ER matrix pellets formulations

Formulation Code	Angle of Repose	Bulk Density	Tapped Density	Compressibility Index	Hausner Ratio	Moisture Content	Yield	Friability	Assay
	θ	g/ml	g/ml	%		%	%	%	%
F1	21.324	0.666	0.714	6.723	1.072	18.591	88.641	0.523	96.163
F2	21.697	0.689	0.741	6.992	1.075	15.143	84.971	0.608	99.741
F3	20.582	0.652	0.704	7.386	1.080	9.947	74.698	0.589	97.957
F4	25.639	0.747	0.807	7.435	1.080	25.667	79.614	0.399	96.745
F5	24.714	0.752	0.821	8.404	1.092	31.357	74.018	0.408	98.237
F6	25.916	0.789	0.859	8.149	1.089	35.987	69.943	0.418	100.024
F7	25.146	0.799	0.876	8.790	1.096	21.228	85.472	0.476	99.242
F8	26.357	0.765	0.832	8.053	1.088	18.298	84.608	0.287	97.589
F9	28.699	0.760	0.834	8.873	1.097	13.952	90.947	0.591	98.241
F10	37.473	0.839	0.999	16.016	1.191	13.694	88.364	0.340	99.415
F11	38.258	0.801	0.989	19.009	1.235	11.037	91.348	0.379	99.876
F12	40.981	0.779	0.975	20.103	1.252	9.024	94.289	0.251	98.279
F13	25.007	0.671	0.710	5.493	1.058	29.195	76.617	0.308	99.873
F14	27.242	0.606	0.648	6.481	1.069	24.834	81.753	0.291	100.390
F15	26.321	0.669	0.719	6.954	1.075	19.479	85.439	0.258	99.220
F16	25.364	0.769	0.831	7.461	1.081	5.870	92.760	0.578	97.289
F17	26.821	0.785	0.861	8.827	1.097	3.930	96.219	0.530	99.657
F18	28.974	0.815	0.906	10.044	1.112	2.966	97.124	0.599	98.917
F19	32.147	0.826	0.931	11.278	1.127	5.942	94.283	0.276	99.814
F20	34.917	0.839	0.964	12.967	1.149	2.456	97.068	0.342	97.668
F21	25.231	0.654	0.713	8.275	1.090	27.894	87.925	0.562	98.749
F22	27.951	0.678	0.730	7.123	1.077	24.276	91.478	0.431	98.529
F23	28.367	0.651	0.710	8.310	1.091	19.875	83.879	0.539	99.384
F24	34.852	0.775	0.891	13.019	1.150	21.763	86.168	0.487	99.876
F25	32.753	0.801	0.939	14.696	1.172	18.258	87.309	0.509	98.254
F26	34.258	0.794	0.939	15.442	1.183	15.734	90.951	0.417	97.413
F27	31.159	0.701	0.798	12.155	1.138	26.013	79.571	0.378	99.643
F28	31.984	0.755	0.879	14.107	1.164	9.876	96.527	0.314	98.872
F29	32.679	0.721	0.848	14.976	1.176	10.325	94.994	0.291	100.954
F30	32.774	0.745	0.852	12.559	1.144	7.410	90.890	0.451	98.371
F31	33.891	0.762	0.885	13.898	1.161	4.337	95.840	0.514	100.547
F32	33.694	0.800	0.937	14.621	1.171	4.230	88.197	0.479	99.875

### Assay and moisture content of Meclizine HCl pellets

The content (percent) of Meclizine HCl in each pellet formulation was within the label claim (60 mg/capsule) as shown in Table 3. MC of all formulations was decreased with the increase in concentration of lipids (Table 3). Among all four lipids, the least MC was observed in CW pellets whereas the highest MC was noted in Precirol^®^ pellets.

### Effect of dissolution medium pH on drug release

**Fig. 4:**
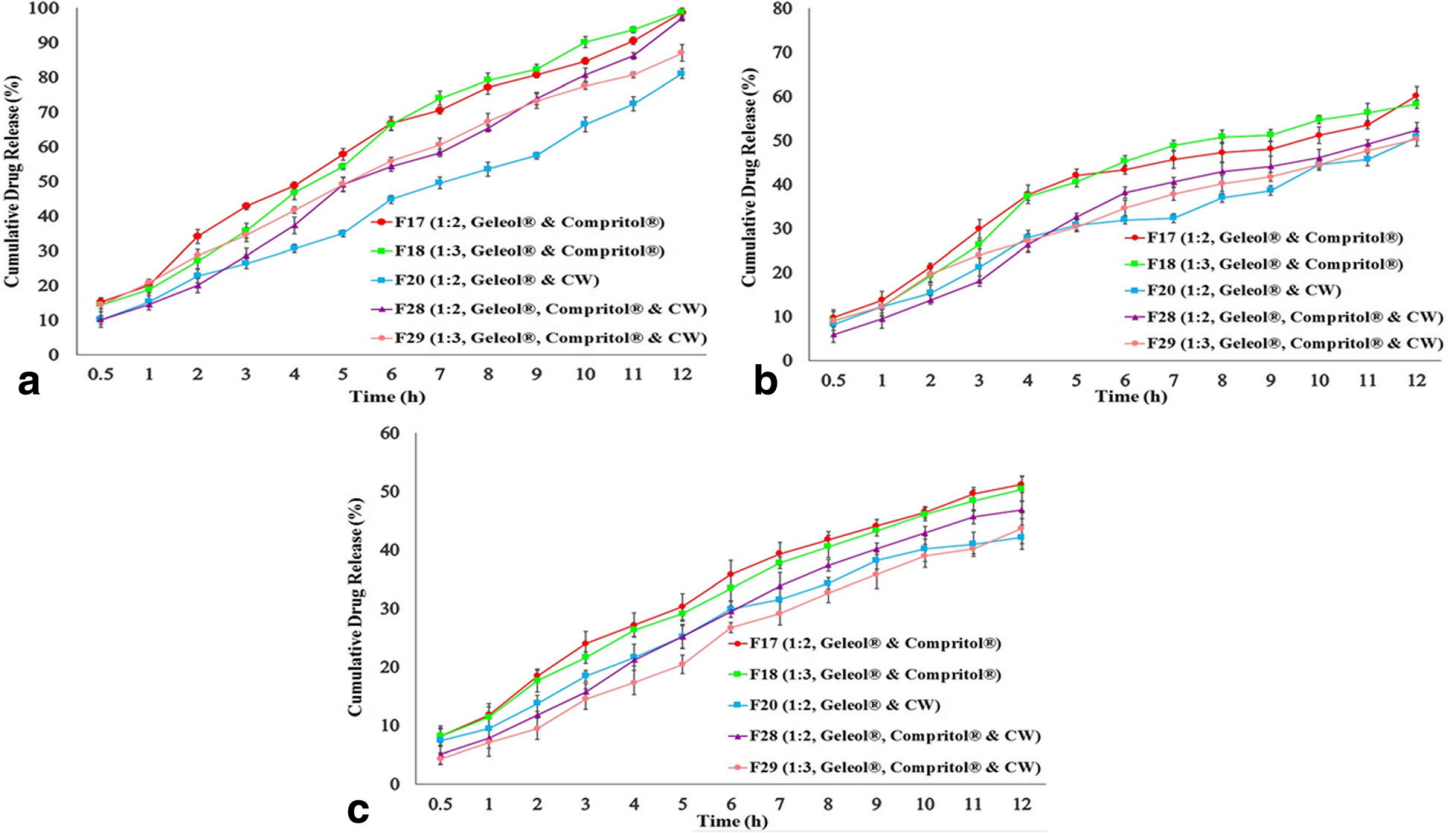
In vitro release profiles showing combinations of Geleol^®^ and Compritol^®^ in drug/lipid ratio of 1:2 (F17), 1:3 (F18), combination of Geleol^®^ and CW in drug/lipid ratio of 1:2 (F20) and combination of Geleol^®^, Compritol^®^ and CW in drug/lipid ratio of 1:2 (F28), and 1:3 (F29) at (**a**) pH 1.2 (**b**) pH 4.5 (**c**) pH 6.8

Fig. 4(a), (b) & (c) show dissolution profiles of Meclizine HCl pellets at pH 1.2 (HCl), 4.5 and 6.8 (phosphate buffer) respectively. Meclizine HCl release was decreased as pH increased from 1.2 to 6.8.

## Drug release kinetics

### Model-dependent methods

Table 4 shows release kinetic data of all formulations. On the basis of best goodness of fit, the most appropriate model was selected. Optimized formulations containing combination of Geleol^®^ and Compritol^®^ at drug/lipid ratio of 1:2 (F17) and 1:3 (F18) showed highest linearity when applied to Korsmeyer-Peppas equation (R^2^ = 0.978–0.993). These formulations exhibited non-Fickian diffusion (anomalous transport), indicating that Meclizine HCl release was controlled by both diffusion and erosion.

**Table 4:** Kinetic parameters for dissolution data of different Meclizine HCl ER matrix pellets formulations according to various kinetic models

Codes	Zero order		First order		Higuchi model		Hixson-Crowell model		Baker and Lonsdale model		Jander’s equation model		Korsmeyer-Peppas model			MDT	DE_6_
	R2	K0 (h-1)	R2	K1 (h-1)	R2	KH (h-1/2)	R2	KHC (h-1)	R2	KBL (h-1)	R2	KJ (h-1/2)	R2	KKP (h-n)	n	h	%
F1	0.917	16.152	0.937	1.992	0.972	45.274	0.977	1.142	0.974	0.197	0.925	0.504	0.986	0.507	0.490	1.724	N/A
F2	0.964	18.114	0.954	0.225	0.990	45.081	0.995	1.012	0.993	0.165	0.966	0.771	0.993	0.620	0.425	0.819	N/A
F3	0.987	29.600	0.961	3.243	1.000	64.028	0.988	1.756	0.983	0.256	0.961	1.028	1.000	0.724	0.451	0.687	N/A
F8	0.998	25.444	0.954	2.204	0.983	54.270	0.973	1.353	0.966	0.250	0.935	0.840	0.972	0.701	0.536	0.531	N/A
F9	0.991	12.015	0.809	1.515	0.973	40.471	0.921	0.461	0.866	0.116	0.830	0.608	0.985	0.323	0.575	1.343	N/A
F11	0.997	21.762	0.961	2.611	0.992	53.325	0.983	0.665	0.948	0.205	0.948	0.600	0.997	0.528	0.572	1.496	N/A
F12	0.992	5.237	0.981	0.136	0.982	22.473	0.991	0.124	0.939	0.016	0.944	0.222	0.991	0.185	0.556	4.884	29.676
F14	0.958	20.305	0.986	2.029	0.979	44.307	1.000	1.355	1.000	0.205	0.988	0.623	0.995	0.809	0.281	0.528	N/A
F15	0.988	17.162	0.852	1.342	0.980	41.982	0.921	0.948	0.911	0.139	0.861	0.485	0.978	0.692	0.323	0.921	N/A
F16	0.957	15.413	0.908	2.089	0.989	42.662	0.978	0.760	0.973	0.155	0.936	0.686	0.990	0.481	0.519	1.159	NA
F17	0.978	7.315	0.916	0.515	0.989	31.730	0.977	0.230	0.924	0.066	0.914	0.503	0.993	0.244	0.551	4.836	33.933
F18	0.974	7.697	0.957	0.404	0.979	33.287	0.989	0.235	0.947	0.041	0.930	0.416	0.978	0.215	0.597	4.832	33.698
F19	0.995	30.747	0.954	2.650	1.000	66.247	0.982	1.786	0.975	0.259	0.950	0.813	0.999	0.719	0.461	0.700	NA
F20	0.995	5.366	0.968	0.150	0.956	22.691	0.982	0.120	0.905	0.014	0.913	0.204	0.978	0.166	0.578	5.448	27.074
F22	0.887	17.594	0.663	3.561	0.841	46.653	0.740	0.748	0.682	0.356	0.654	1.950	0.905	0.472	0.533	1.921	NA
F23	0.967	14.414	0.796	2.389	0.966	42.713	0.907	0.606	0.872	0.195	0.832	1.206	0.963	2.389	0.501	1.855	NA
F24	0.990	24.496	0.966	2.309	1.000	52.932	0.990	1.546	0.987	0.230	0.963	0.706	1.000	0.777	0.345	0.604	NA
F25	0.960	14.995	0.874	1.205	0.971	37.046	0.933	0.858	0.929	0.128	0.886	0.442	0.973	0.723	0.273	0.816	NA
F26	0.950	13.089	0.908	0.635	0.987	36.336	0.978	0.701	0.979	0.103	0.940	0.315	0.992	0.637	0.316	1.023	NA
F27	0.875	16.587	0.966	2.049	0.937	42.169	0.988	0.962	0.993	0.115	0.985	0.377	0.953	0.559	0.517	0.728	NA
F28	0.991	7.284	0.842	0.937	0.959	30.910	0.927	0.201	0.802	0.109	0.839	0.310	0.984	0.166	0.696	6.008	26.679
F29	0.991	5.791	0.985	0.148	0.988	24.933	0.995	0.143	0.948	0.022	0.950	0.207	0.997	0.146	0.675	4.835	31.384
F30	1.000	36.837	1.000	5.368	1.000	62.856	1.000	3.197	1.000	0.479	1.000	1.176	1.000	0.987	0.298	0.343	N/A
F31	0.959	15.883	0.964	1.980	0.982	39.478	0.994	0.960	0.993	0.153	0.967	0.678	0.982	0.645	0.389	0.743	N/A
F32	0.965	9.731	0.820	2.149	0.993	31.476	0.938	0.448	0.929	0.163	0.880	0.539	0.995	0.508	0.371	1.724	N/A

“R2 “is the regression coefficient; “K” is the release rate constant for respective models; “n” is the diffusion exponent; “MDT” is the mean dissolution time, “DE” is the dissolution efficiency at 6 h. N/A indicates that DE is not calculated as 90% drug released before 6 h. F4, F5, F6, F7, F10, F13 and F21 released 90% drug within 2 h

Combination of CW either with Geleol^®^ (F20) or Geleol^®^ and Compritol^®^ (F28 and F29) displayed best fit in Zero-order (R^2^ = 0.991–0.995) indicating concentration independent Meclizine HCl release. The release constants (*k*) were higher for increased drug mass fraction and lower for increased lipid concentration. Concerning the influence of lipid type, lower release constants were found in CW pellets.

MDT was directly related to physicochemical properties of drug as well as the concentration and nature of lipids [19]. MDT was increased with increased concentration of lipids. This effect was more pronounced in combinations of Geleol^®^ and CW (F20) and Geleol^®^, Compritol^®^ and CW (F28). The highest DE_6_ was observed in combination of Geleol^®^ and Compritol^®^ (F17).

### Model-independent method

Out of thirty two formulations, five formulations F17 (Geleol^®^ and Compritol^®^, 1:2), F18 (Geleol^®^ and Compritol^®^, 1:3), F20 (Geleol^®^ and CW, 1:2), F28 (Geleol^®^, Compritol^®^ and CW, 1:2) and F29 (Geleol^®^, Compritol^®^ and CW, 1:3) showed extended drug release up to 12 h. Combination of Geleol^®^ and Compritol^®^ in drug/lipid ratio of 1:2 (F17) was selected as a reference formulation because of high sphericity of pellets with smooth surface and controlled release profile. Although, similar properties were obtained in combination of Geleol^®^ and Compritol^®^ pellets in drug/lipid ratio of 1:3 (F18), but higher quantity of Compritol^®^ was used in F18 as compared to F17. Dissolution profile of F18 was only similar with F17 having *f*_2_ value 71.604.

### Scanning electron microscopy (SEM)

**Fig. 5:**
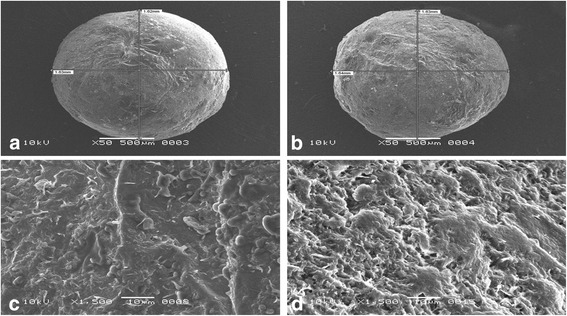
SEM images showing combinations of Geleol^®^ and Compritol^®^ in drug/lipid ratio of (**a**) 1:2 (F17) (**b**) 1:3 (F18) (**c**) cross section of F17 (1:2) (**d**) cross section of F18 (1:3)

**Fig. 6:**
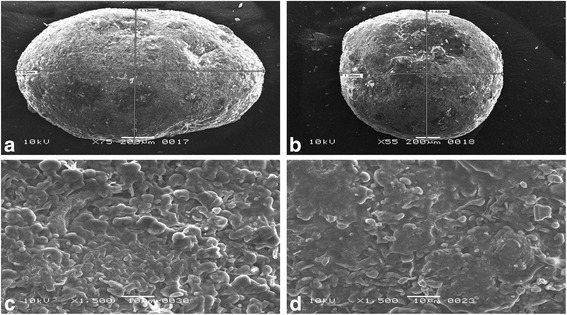
SEM images showing (**a**) combination of Geleol^®^ and CW in drug/lipid ratio of 1:2 (F20) (**b**) combination of Geleol^®^, Compritol^®^ and CW in drug/lipid ratio of 1:2 (F28) (**c**) cross section of F20 (**d**) cross section of F28

Surface morphology of pellets was revealed by SEM. Combinations of Geleol^®^ and Compritol^®^ F17 and F18 exhibited smooth surface and appeared spherical and intact in shape, shown in Fig. 5(a) & (b) respectively. Geleol^®^ and Compritol^®^ pellets in drug/lipid ratio of 1:2 (F17), was compared with drug/lipid ratio of 1:3 (F18), for external morphology and texture. These (Fig. 5(a) & (b)) show that both pellet formulations were almost similar in appearance. SEM images indicated that external morphology was independent of drug/lipid ratio. Significant difference in cross section of drug/lipid ratio of 1:2 (F17) and 1:3 (F18) was observed. Figure 5(d) shows that F18 (1:3) had dense network of lipid matrix, as compared to F17 (1:2), shown in Fig. 5(c). SEM images of Geleol^®^ and CW (F20) displayed irregularly shaped pellets with highly rough surface and hollow depressions, shown in Fig. 6(a). These irregularly shaped pellets were made almost spherical by the addition of Compritol^®^ in Geleol^®^ and CW pellets (F28), illustrated in SEM image Fig. 6(b). It showed rough surface with less depressions and were nearly spherical in shape. Figure 6c & d show cross section of F20 and F28 respectively.

### Energy dispersive spectroscopy (EDS)

**Fig. 7:**
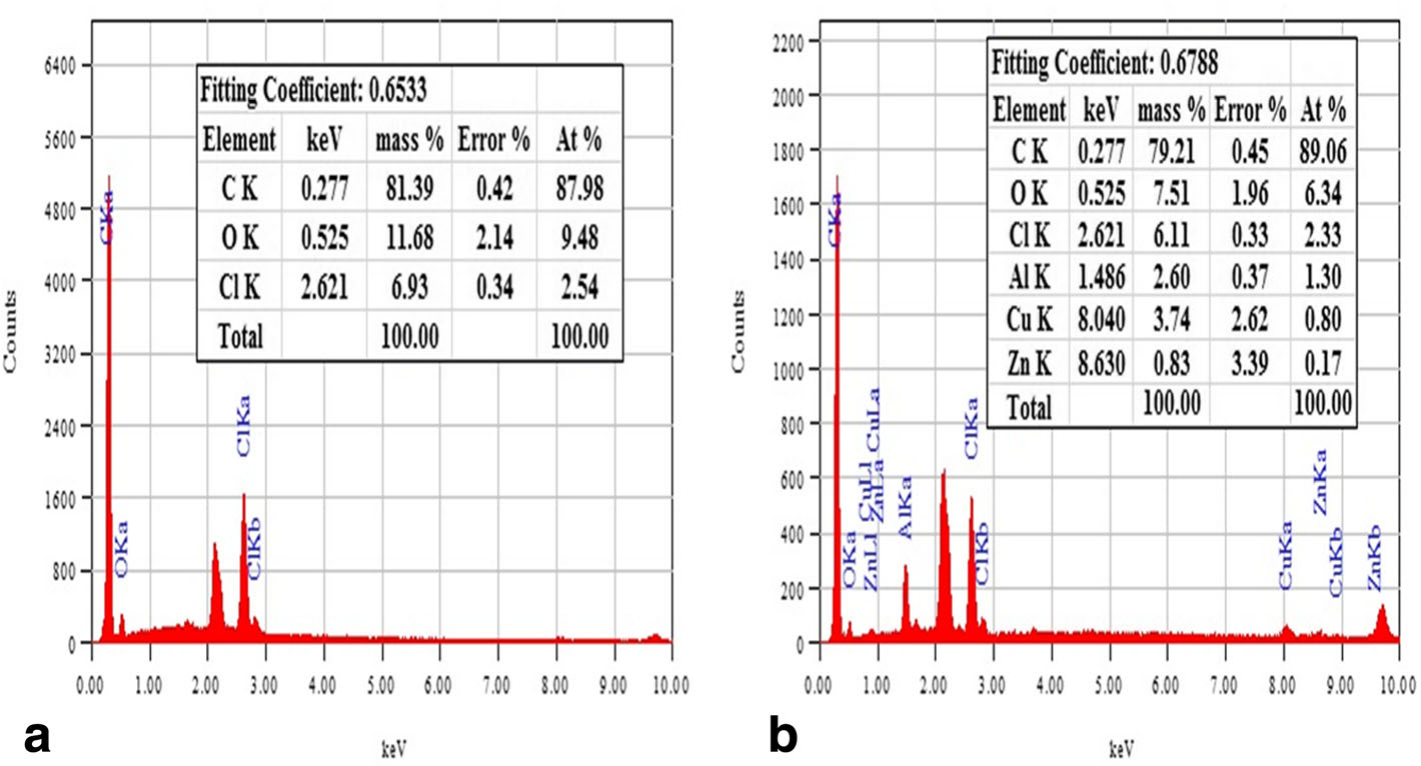
EDS spectra showing (**a**) combination of Geleol^®^ and Compritol^®^ (F17) (**b**) combination of Geleol^®^, Compritol^®^ and CW (F28)

Elements carbon, oxygen and chlorine were found in combination of Geleol^®^ and Compritol^®^ pellets (F17) as shown in Fig. 7(a). The highest content of carbon was due to the organic drug containing carbon chain. Combination of Geleol^®^, Compritol^®^ and CW (F28) showed additional peaks of aluminum, copper and zinc (Fig. 7(b)) due to presence of CW in addition to Compritol^®^ and Geleol^®^.

### Stability

Optimized formulations of lipids showed acceptable stability for 6 months at 40 °C ± 2 °C/ 75% ± 5% RH. No significant difference in physical appearance, in vitro drug release and drug content was observed except in the combination of Geleol^®^ and CW (F20). This lipid combination released only 66% drug at the end of 12 h after storage of 6 months. Results indicated shelf-life of 37 months with 90% lower acceptance limit of label claim.

## Discussion

Extended release pellets of poorly soluble antiemetic agent Meclizine HCl were prepared by extrusion spheronization method using Geleol^®^, Precirol^®^, Compritol^®^ and CW. Highly spherical pellets with faster drug release were prepared using Geleol^®^. These pellets were rapidly hydrated in dissolution medium due to presence of two free hydroxyl groups in their structure and a higher HLB value (3.8), as higher HLB values are associated with increased drug release rate [32]. Similar surface active property of GMS was observed by Quadir et al. [22]. It is also reported that addition of GMS in pellet formulations resulted in higher drug release rate due to formation of pores [11]. Wadher et al., formulated GMS based SR tablets of Metformin HCl in drug/wax ratio of 1:0.6 which released 95% drug at the end of 12 h [33]. Cheboyina and Wyandt reported that GMS pellets reduced drug release rate depending on drug solubility [10].

ER Meclizine HCl pellets with Precirol^®^ showed rapid drug release. Pellet size was influenced by the physical properties of the blend constituents, drug/lipid ratio, type and quantity of granulating fluid. It was observed that Precirol^®^ used in drug/lipid ratio of 1:3 (60%) resulted in complete drug released within 2 hours. It was previously reported that Precirol^®^ was unable to sustain the drug release up to 12 h [34]. On the contrary, Pongjanyakul et al., reported that the drug release was decreased with increased proportions of GPS [7].

Release of Meclizine HCl from Compritol^®^ pellets was decreased with increase in concentration of Compritol^®^. This release retardant effect was due to the presence of longer fatty acid chain length of Behenic acid (C22), providing higher degree of lipophilicity [32]. However, it was observed that Compritol^®^ failed to extend Meclizine HCl release up to 12 h which is in line with findings reported by Gu et al. [34].

CW is highly hydrophobic in nature with low wettability [19]. It controlled drug release extensively and imparted the highest retardation effect among the four lipids. CW is multi-constituent, containing alkyl esters of wax acids (80%), particularly myricyl cerotate, free monohydric alcohols (10%), resin and lactose [22]. In design of Meclizine HCl ER pellets, CW acted as effective release retardant. Since matrix pellets were devoid of channelling agents, absence of pores and cracks further inhibited drug release [19]. Although, it was reported in previous studies that ground CW (4–20%) sustained release of theophylline for 3 h [5] and CW (25%) resulted in extensive burst release within 1 h [22].

MCC is a well-known extrusion spheronization aid as it holds water like a sponge and inhibits separation of water from the solid blend during processing. When used with CW, MCC formed irregularly shaped pellets because irregular particles of CW were distributed unevenly, causing hindrance during spheronization. Similar finding was also observed by Singh et al. [5]. MCC based pellets prolonged the drug release because they do not disintegrate. This decreases bioavailability of low aqueous soluble drugs [35]. However, release of poorly soluble Meclizine HCl was unaffected by addition of 40% MCC.

The release profile of Meclizine HCl from each lipid was significantly different, indicating marked influence of physicochemical composition of lipids on drug release rate. It is clearly evident from the results of Group 1 that drug/lipid ratio of 1:0.5–1:1 for all lipids was insufficient for sustaining the Meclizine HCl release. Therefore, drug/lipid ratio was increased up to 1:3 in further groups to achieve the desirable extended release profile of Meclizine HCl. Poor drug retardation effect in preparation of SR Milnacipran HCl was reported by Parjiya et al., when drug/lipid ratio was fixed at 1:1 during initial screening of Stearic acid, CW, Compritol^®^ and Bees wax, therefore, drug/wax ratio was increased from 1:1 to 1:1.25, 1:1.5 and 1:1.75. [24].

Irrespective of lipid type, its amount had an inverse influence on drug release i.e. rate of Meclizine HCl release decreased with higher level of lipids present in extruded-spheronized pellets with exception of Geleol^®^. This might be due to the slower penetration of dissolution medium in matrices as a result of the increased lipophilicity of waxy substances [33]. Lipid content dependent drug release was more significant in case of CW as compared to others. CW is more lipophilic matrix that hardly allows water to penetrate into the pores of the matrix. It contains 5% resins, higher amount of fatty esters and lower hydroxyl number and free fatty acids resulting in reduced dissolution in acidic medium [20]. Geleol^®^ is more prone to hydration in dissolution medium because of hydroxyl groups [10].

Three combinations of lipids: (i) Geleol^®^ and Compritol^®^ (ii) Geleol^®^ and CW (iii) Geleol^®^, Compritol^®^ and CW in different groups displayed extended release of antiemetic agent-Meclizine HCl up to 12 h with acceptable sphericity. In addition to MCC, Geleol^®^ was also added in each combination as it formed highly spherical pellets of Meclizine HCl. Geleol^®^ and Compritol^®^ pellets in drug/lipid ratio of 1:2–1:3 presented desirable characteristics and extended drug release up to 12 h with the formation of smooth surface round pellets. The tortuosity of the matrix and drug diffusion path length were increased by combination of lipids and increment in lipid content, thus, reduced the diffusion and erosion from matrix. A similar observation is documented by Wadher et al., that release of metformin hydrochloride was more strongly retarded, when formulated with combination of waxes compared to metformin HCl formulation with single wax content because higher lipophilicity was observed in combination of waxes [33].

In sustained release dosage forms, Compritol^®^ is considered as an ideal excipient to substitute hydrophilic matrix, since, it is not associated with alcohol related dose dumping. It is highly resistant to physiological conditions (pH, digestion) and reduces burst effect of highly water soluble drugs [17]. Compritol^®^ provides stable release profile during storage [14]. Similar findings were noted by Jagdale et al., showing that Compritol^®^ is a mixture of mono (18%), di (52%) and tri (28%) behenates of glycerol and is especially designed to produce sustained release of drugs which cannot be obtained from pure di or triglycerides [36].

This study shows that Geleol^®^ and CW pellets excessively retarded the release of Meclizine HCl in comparison to Geleol^®^ and Compritol^®^ pellets. Compritol^®^ has increased wettability in dissolution medium owing to its non-ionic surfactant characteristic with hydroxyl number 102.6 [20]. In CW matrix system, complete drug release is impossible because impermeable wax film entrap some fraction of dose [19].

Shape descriptors like aspect ratio and two dimensional shape factor were measured for single lipid and combinations of lipids extruded-spheronized pellets to analyse impact of lipid type, lipid amount and lipid combination on shape of Meclizine HCl pellets. Geleol^®^ formed highly spherical pellets of Meclizine HCl indicated by larger values of aspect ratio and two dimensional shape factor. Two dimensional shape factor was increased with greater amount of Geleol^®^ used in pellets. Precirol^®^ and Compritol^®^ formed spherical pellets with acceptable aspect ratio and shape factors. Insignificant differences in aspect ratio of Precirol^®^ and Compritol^®^ pellets were observed when compared with Geleol^®^. However, lower two dimensional shape factors indicated that Precirol^®^ and Compritol^®^ pellets were not perfectly round like Geleol^®^ pellets. Two dimensional shape factor is more sensitive to surface irregularities and to deviations from the ideal round shape as compared to aspect ratio [27]. Aspect ratio and two dimensional shape factor of CW pellets were beyond the acceptable lower limit indicating formation of irregular pellets. Two dimensional shape factor was decreased with increased content of CW indicating uneven surface of pellets. Geleol^®^ had the highest shape factors whereas, CW had the lowest shape factors, among all four lipids utilized for preparation of pellets. On the basis of findings of Group 1, Geleol^®^ was added in each lipid combination which resulted in acceptable shape descriptors.

SEM images further confirmed findings of stereomicroscopy. Combination of Geleol^®^ and Compritol^®^ indicated formation of spherical and intact pellets with smooth surfaces. Sphericity and surface smoothness of this lipid combination were independent of drug/lipid ratio. Higher lipid content formed dense network of lipid matrix. Combination of Geleol^®^ and CW formed highly rough pellets. Presence of Geleol^®^ failed to improve the surface roughness of CW pellets, clearly evident in SEM images. Irregular particles of CW were distributed unevenly which caused interruption during spheronization [5]. This surface roughness of CW was reduced by combination of Geleol^®^, Compritol^®^ and CW in drug/lipid ratio of 1:2 (F28) and 1:3 (F29). SEM is coupled with EDS which is used to characterize elements in situ. In comparison to combination of Geleol^®^ and Compritol^®^ combination of Geleol^®^, Compritol^®^ and CW showed additional peaks of aluminium, copper and zinc. These additional peaks may be due to vegetable origin of CW which is a natural ester lipid, obtained by extraction of carnauba palm [18]. This combination indicated presence of less oxygen when compared to combination of Geleol^®^ and Compritol^®^ which confirm the reason of utilization of less granulating fluid during wet massing.

Meclizine HCl pellets were evaluated in different dissolution medium and pH dependent drug release was observed. Meclizine HCl is acidic salt of weakly basic drug having pKa 6.12. Therefore, its solubility and ionization are reduced at alkaline pH and increased at acidic pH. This may be due to the conversion of the hydrochloride salt to its less soluble free base [15]. Similar pH dependent Meclizine HCl release was also observed by Mahrous et al. [2].

Release of Meclizine HCl from Compritol^®^ pellets was best described by Korsmeyer-Peppas model indicating non-Fickian diffusion. Initially, Meclizine HCl was dissolved from external surface of pellets causing formation of pores in matrix. The matrix became soft with progressive dissolution leading to erosions, formation of channels and promoting penetration of medium to dissolve the drug. This dissolved drug diffused through the channels into the medium. This finding is consistent with previously reported studies [32, 33, 36, 37]. The value of *n* is dependent on type of lipid and physicochemical properties of drug. CW pellets showed concentration independent release of Meclizine HCl. However, diffusion [19] and Fickian mechanism [20] associated with the use of CW matrices were also reported.

## Conclusions

Single lipid matrix extruded-spheronized pellets of Geleol^®^, Precirol^®^ and Compritol^®^ failed to extend Meclizine HCl release up to 12 h, even in drug/lipid ratio of 1:2. Although the release of Meclizine HCl was extended up to 12 h with CW, but the pellets were irregularly shaped. This irregularity in shape was effectively controlled by addition of Geleol^®^. Matrix extruded-spheronized pellets prepared with blends of (i) Geleol^®^ and Compritol^®^, (ii) Geleol^®^ and CW (iii) Geleol^®^, Compritol^®^ and CW successfully extended release of Meclizine HCl up to 12 h. These lipids combinations in ratio of 1:2 can be effectively used to prepare ER matrix pellets of Meclizine HCl. Lipid combination of Geleol^®^ and Compritol^®^ (F17) formed highly spherical pellets with smooth surfaces and successfully sustained the release of Meclizine HCl up to 12 h. These lipids combinations can be effectively employed to design extended release pellet formulation of Meclizine HCl by extrusion spheronizaton technique, for the control of vertigo, pruritus, nausea and dizziness up to extended period of time.

## Abbreviations

ASTM: American Society of Testing and Materials
CW: Carnauba Wax
DE: Dissolution Efficiency
ER: Extended Release
GB: Glyceryl behenate
GMS: Glyceryl monostearate
GPS: Glyceryl palmito Stearate
HCl: Hydrochloric Acid
ICH: International Conference on Harmonization
MC: Moisture Content
MCC: Microcrystalline Cellulose
MDT: Mean Dissolution Time
RH: Relative Humidity
SR: Sustained Release

## References

[1] Wang Z, Lee B, Pearce D, Qian S, Wang Y, Zhang Q, Chow MS (2012). Meclizine metabolism and pharmacokinetics: formulation on its absorption. J Clin Pharmacol.

[2] Mahrous GM, Shazly GA, Ibrahim MA (2011). Formulation and evaluation of meclizine HCl orally disintegrating tablets. Bull Pharm Sci.

[3] Symphonyhealth: http://symphonyhealth.com/wp-content/uploads/2015/05/Top-200-Drugs-of-2014.pdf (accessed: 25.03.17)2017.

[4] PfizerProductsAntivert: https://www.pfizerpro.com/pfizer-products. (accessed: 25.03.17) 2017.

[5] Singh R, Poddar S, Chivate A (2007). Sintering of wax for controlling release from pellets. AAPS PharmSciTech.

[6] Kranz H, Jürgens K, Pinier M, Siepmann J (2009). Drug release from MCC-and carrageenan-based pellets: experiment and theory. Eur J Pharm Biopharm.

[7] Pongjanyakul T, Medlicott NJ, Tucker IG (2004). Melted glyceryl palmitostearate (GPS) pellets for protein delivery. Int J Pharm.

[8] Hamdani J, Moës AJ, Amighi K (2003). Physical and thermal characterisation of Precirol® and Compritol® as lipophilic glycerides used for the preparation of controlled-release matrix pellets. Int J Pharm.

[9] Cheboyina S, Chambliss WG, Wyandt CM (2004). A novel freeze Pelletization technique for preparing Matix pellets. Pharm Technol.

[10] Cheboyina S, Wyandt CM (2008). Wax-based sustained release matrix pellets prepared by a novel freeze pelletization technique: II. In vitro drug release studies and release mechanisms. Int J Pharm.

[11] Roblegg E, Jäger E, Hodzic A, Koscher G, Mohr S, Zimmer A, Khinast J (2011). Development of sustained-release lipophilic calcium stearate pellets via hot melt extrusion. Eur J Pharm Biopharm.

[12] Rahman MA, Ahuja A, Baboota S, Bali V, Saigal N, Ali J (2009). Recent advances in pelletization technique for oral drug delivery: a review. Current drug delivery.

[13] Nasiri MI, Yousuf RI, Shoaib MH, Fayyaz M, Qazi F, Ahmed K. Investigation on release of highly water soluble drug from matrix-coated pellets prepared by extrusion–spheronization technique. J Coat Technol Res. 2016:1–12.

[14] Becker K, Salar-Behzadi S, Zimmer A (2015). Solvent-free melting techniques for the preparation of lipid-based solid oral formulations. Pharm Res.

[15] Gao Z, Yu L, Clark S, Trehy M, Moore T, Westenberger B, Buhse L, Kauffman J, Bishop B, Velazquez L (2015). Dissolution Testing for bioavailability of over-the-counter (OTC) drugs—a technical note. AAPS PharmSciTech.

[16] Shukla D, Chakraborty S, Singh S, Mishra B (2011). Lipid-based oral multiparticulate formulations–advantages, technological advances and industrial applications. Expert opinion on drug delivery.

[17] GattefosseProducts: http://www.gattefosse.com/en/applications/?administration-route,oral,substained-release. (accessed:25.03.17) 2017.

[18] Pezzini BR, Grossl AD, Muraro A, Bazzo GC, Soares L (2014). Formulation and in vitro assessment of sustained release matrix tablets of atenolol containing Kollidon SR and carnauba wax. Afr J Pharm Pharmacol.

[19] Reza MS, Quadir MA, Haider SS (2003). Comparative evaluation of plastic, hydrophobic and hydrophilic polymers as matrices for controlled-release drug delivery. J Pharm Pharm Sci.

[20] Özyazıcı M, Gökçe EH, Ertan G (2006). Release and diffusional modeling of metronidazole lipid matrices. Eur J Pharm Biopharm.

[21] Reddy KR, Mutalik S, Reddy S (2003). Once-daily sustained-release matrix tablets of nicorandil: formulation and in vitro evaluation. AAPS PharmSciTech.

[22] Quadir MA, Rahman MS, Karim MZ, Akter S, Awkat M, Reza M (2003). Evaluation of hydrophobic materials as matrices for controlled-release drug delivery. Pak J Pharm Sci.

[23] Wilson B, Babubhai PP, Sajeev MS, Jenita JL, Priyadarshini BSR (2013). Sustained release enteric coated tablets of pentaprazole: formulation, in vitro and in vivo evaluation. Acta Pharma.

[24] Parejiya PB, Barot BS, Patel HK, Mehta DM, Shelat PK, Shukla A (2014). Release modulation of highly water soluble drug using solid dispersion: impact of dispersion and its compressed unit. Journal of Pharmaceutical Investigation.

[25] USP 38 NF 33: U.S. Pharmacopeia National Formulary. Rockville: The United States Pharmacopeial Convention; 2015.

[26] Chamsai B, Sriamornsak P (2013). Novel disintegrating microcrystalline cellulose pellets with improved drug dissolution performance. Powder Technol.

[27] Podczeck F, Rahman S, Newton J (1999). Evaluation of a standardised procedure to assess the shape of pellets using image analysis. Int J Pharm.

[28] Kitak T, Govedarica B, Srčič S (2013). Physical properties of pharmaceutical pellets. Chem Eng Sci.

[29] FDADissolution: http://www.accessdata.fda.gov/scripts/cder/dissolution/index.cfm (accessed 11.03.16). 2016.

[30] Qazi F, Shoaib MH, Yousuf RI, Qazi TM, Mehmood ZA, Hasan SMF (2013). Formulation development and evaluation of Diltiazem HCl sustained release matrix tablets using HPMC K4M and K100M. Pak J Pharm Sci.

[31] Costa P, Lobo JMS (2001). Modeling and comparison of dissolution profiles. Eur J Pharm Sci.

[32] Abd-Elbary A, Tadros MI, Alaa-Eldin AA (2013). Sucrose stearate-enriched lipid matrix tablets of etodolac: modulation of drug release, diffusional modeling and structure elucidation studies. AAPS PharmSciTech.

[33] Wadher KJ, Kakde RB, Umekar MJ (2010). Formulations of sustained release metformin hydrochloride tablet using combination of lipophilic waxes by melt granulation technique. Afr J Pharm Pharmacol.

[34] Gu X, Fediuk DJ, Simons FER, Simons KJ (2004). Evaluation and comparison of five matrix excipients for the controlled release of acrivastine and pseudoephedrine. Drug Dev Ind Pharm.

[35] Kranz H, Jurgens K, Pinier M, Siepmann J (2009). Drug release from MCC- and carrageenan-based pellets: experiment and theory. Eur J Pharm Biopharm.

[36] Jagdale S, Patil S, Kuchekar B, Chabukswar A (2011). Preparation and characterization of Metformin hydrochloride− Compritol 888 ATO solid dispersion. J Young Pharm.

[37] Yan X, He H, Meng J, Zhang C, Hong M, Tang X (2012). Preparation of lipid aspirin sustained-release pellets by solvent-free extrusion/spheronization and an investigation of their stability. Drug Dev Ind Pharm.

